# Sub‐maximal aerobic exercise training reduces haematocrit and ameliorates symptoms in Andean highlanders with chronic mountain sickness

**DOI:** 10.1113/EP089975

**Published:** 2021-09-30

**Authors:** José Luis Macarlupú, Gustavo Vizcardo‐Galindo, Rómulo Figueroa‐Mujíca, Nicolas Voituron, Jean‐Paul Richalet, Francisco C. Villafuerte

**Affiliations:** ^1^ Laboratorio de Fisiología Comparada Facultad de Ciencias y Filosofía Universidad Peruana Cayetano Heredia Lima Perú; ^2^ Instituto de Investigaciones de la Altura (IIA) Universidad Peruana Cayetano Heredia Lima Perú; ^3^ Laboratoire Hypoxie et Poumon UMR INSERM U1272 Université Sorbonne Paris Nord Bobigny France; ^4^ Laboratory of Excellence GReX Paris France; ^5^ Département STAPS Université Sorbonne Paris Nord Bobigny France

**Keywords:** Andean highlanders, chronic mountain sickness, excessive erythrocytosis, exercise training, high altitude, Monge´s disease

## Abstract

**New Findings:**

- **What is the central question of this study?**
  What is the effect of sub‐maximal aerobic exercise training on signs and symptoms of chronic mountain sickness (CMS) in Andean highlanders?
- **What is the main finding and its importance?**
  Aerobic exercise training (ET) effectively reduces haematocrit, ameliorates symptoms and improves aerobic capacity in CMS patients, suggesting that a regular aerobic ET programme might be used as a low‐cost non‐invasive/non‐pharmacological management strategy of this syndrome.

**Abstract:**

Excessive erythrocytosis is the hallmark sign of chronic mountain sickness (CMS), a debilitating syndrome associated with neurological symptoms and increased cardiovascular risk. We have shown that unlike sedentary residents at the same altitude, trained individuals maintain haematocrit within sea‐level range, and thus we hypothesise that aerobic exercise training (ET) might reduce excessive haematocrit and ameliorate CMS signs and symptoms. Eight highlander men (38 ± 12 years) with CMS (haematocrit: 70.6 ± 1.9%, CMS score: 8.8 ± 1.4) from Cerro de Pasco, Peru (4340 m) participated in the study. Baseline assessment included haematocrit, CMS score, pulse oximetry, maximal cardiopulmonary exercise testing and in‐office plus 24 h ambulatory blood pressure (BP) monitoring. Blood samples were collected to assess cardiometabolic, erythropoietic, and haemolysis markers. ET consisted of pedalling exercise in a cycloergometer at 60% of ${\dot{V}}_{O_{2}\mathrm{peak}}$ for 1 h/day, 4 days/week for 8 weeks, and participants were assessed at weeks 4 and 8. Haematocrit and CMS score decreased significantly by week 8 (to 65.6 ± 6.6%, and 3.5 ± 0.8, respectively, *P* < 0.05), while ${\dot{V}}_{O_{2}\mathrm{peak}}$ and maximum workload increased with ET (33.8 ± 2.4 vs. 37.2 ± 2.0 ml/min/kg, *P* < 0.05; and 172.5 ± 9.4 vs. 210.0 ± 27.8 W, *P* < 0.01; respectively). Except for an increase in high‐density lipoprotein cholesterol, other blood markers and BP showed no differences. Our results suggest that reduction of haematocrit and CMS symptoms results mainly from haemodilution due to plasma volume expansion rather than to haemolysis. In conclusion, we show that ET can effectively reduce haematocrit, ameliorate symptoms and improve aerobic capacity in CMS patients, suggesting that regular aerobic exercise might be used as a low‐cost non‐invasive and non‐pharmacological management strategy.

## 1 INTRODUCTION

The excessive production of red blood cells (excessive erythrocytosis; EE) is the hallmark feature of chronic mountain sickness (CMS) or Monge's disease, a highly prevalent and incapacitating syndrome in Andean and other high‐altitude populations around the world (Leon‐Velarde et al., 2005). CMS is defined by the presence of EE associated with severe hypoxaemia, neurological sequelae and sleep disorders, also often accompanied by other complications such as pulmonary hypertension and cardio‐cerebrovascular accidents due to adverse changes in blood rheology (Penaloza & Arias‐Stella, 2007; Villafuerte & Corante, 2016). In addition, we and others have shown that EE associates with increased cardiovascular disease risk factors and cardiometabolic disorders such as in‐office and ambulatory hypertension, insulin resistance, dyslipidaemia and metabolic syndrome (Bilo et al., 2020; Corante et al., 2018; De Ferrari et al., 2014; Gonzales & Tapia, 2013; Miele et al., 2016) in various high‐altitude regions across the world (Gonzales & Tapia, 2013; Okumiya et al., 2011, 2010; Sherpa et al., 2011). The long‐term burden of CMS equates to a loss of 3 months of healthy life per year at high altitude (4000 m) (Pei et al., 2012). It is estimated that 5–10% of the world's population living at high‐altitude may develop this condition, and its prevalence increases with altitude and age (Leon‐Velarde et al., 2014; Monge‐C et al., 1989). Above 4300 m in the central Andes of Peru, more than 30% of highlanders by their mid‐50s suffer from EE (Leon‐Velarde et al., 1997; Monge‐C et al., 1992, 1989). CMS signs and symptoms disappear completely when patients descend to sea‐level conditions and following bloodletting or haemodilution at their native altitude of residence, suggesting that the underlying symptoms are secondary to EE. Therefore, treatment strategies for CMS aim to reduce haematocrit and blood viscosity, either by reducing the number of red blood cells or reducing the hypoxic stimulus. However, there is little evidence supporting the safety and efficacy of any long‐term pharmacological or non‐pharmacological option for either and relocation to lower altitudes is unfeasible for the majority of individuals, compromising health, social cohesion and economic wellbeing of patients and their families. Several studies have shown that regular aerobic exercise at 50–60% of maximal effort improves cardio‐respiratory function and O_2_ transport to tissues which might reduce hypoxaemia‐stimulated erythropoiesis (American College of Sports Medicine, 2010; British Thoracic Society Standards of Care Subcommittee on Pulmonary Rehabilitation, 2001; Reis et al., 1997). Moreover, people that practice exercise regularly have lower Hb concentrations than sedentary people (Mairbaurl, 2013; Telford et al., 2003). This difference is even more pronounced in athletes (Bonilla et al., 2005; Lippi & Sanchis‐Gomar, 2019; Schobersberger et al., 1990; Telford et al., 2003).

Studies in Andean dwellers suggest that physical exercise may reduce haematocrit at high altitude. Schmidt et al. (1990) and Cornolo et al. (2005) have shown that native high‐altitude athletes living and training above 4000 m have sea‐level Hb concentrations in contrast to sedentary individuals living at the same altitude. Moreover, a recent study by our group has shown that exercise training (ET) reduces haematocrit in a rat model of chronic hypoxia‐induced erythrocytosis mainly due to exercise‐induced haemolysis without changes in plasma volume (Macarlupu et al., 2021).

Although exercise fatigue and reduced exercise capacity have been commonly reported in CMS patients, allegedly due to systemic or pulmonary haemodynamic burden and O_2_‐diffusion impairment as a consequence of excessive haematocrit and blood viscosity (Letcher et al., 1981; Monge, 1943; Ostergaard, 2020; Pratali et al., 2012; Soria et al., 2019; Stuber et al., 2010; Winslow & Monge‐C, 1987), several studies indirectly suggest that adaptive mechanisms exist to maintain O_2_ transport in the face of a high haematocrit (Juvonen et al., 1991; Lindenfeld et al., 1985). We have previously shown that Andeans diagnosed with mild to moderate CMS can attain normal maximal aerobic capacities compared to healthy Andean controls in Cerro de Pasco (4340 m) (Groepenhoff et al., 2012), and we have also recently confirmed these findings and identified evidence for an adaptive phenotype for O_2_ transport during exercise with EE (Hansen et al., 2021).

These adaptive characteristics challenge the common belief of reduced tolerance to exercise and suggest that CMS highlanders could benefit from regular moderate aerobic ET. Thus we hypothesise that sub‐maximal aerobic ET can reduce excessive haematocrit and ameliorate CMS signs and symptoms. In addition, we hypothesise that ET can improve aerobic capacity and also reduce cardiovascular and cardiometabolic risk factors in CMS highlanders. Therefore, the primary aim of this study was to assess the effect of an 8‐week sub‐maximal aerobic ET programme on haematocrit and CMS score. Additionally we aimed to investigate changes in aerobic capacity, conventional in‐office and ambulatory blood pressure, together with cardiometabolic, erythropoietic and haemolysis blood markers.

## 2 METHODS

### 2.1 Ethical approval

The study was approved by the Institutional Ethics Committee of Universidad Peruana Cayetano Heredia (CIEH‐UPCH approval no. 606‐25‐18, SIDISI no. 100520), and was conducted in accordance with the principles of the *Declaration of Helsinki* (except for registration in a database). All participants received a detailed explanation of the experimental protocol before consent, and were provided with a consent form in Spanish to be signed before participation in the study.

### 2.2 Study participants

Ten untrained men with CMS were recruited for the study, and two of them withdrew before the first session of the ET protocol. Thus, we were able to follow eight CMS patients throughout the study (Table 1). Each participant was their own baseline control. All participants were lifelong residents of Cerro de Pasco, Peru (4340 m). Volunteers were excluded if they had a history of pulmonary, cardiovascular or renal disease; were current smokers; work in mining activities; had undergone surgery, blood transfusions or phlebotomies in the previous 6 months; had travelled to lower altitudes (<3000 m) for more than 7 days during the previous 6 months; or had demonstrated abnormal electrocardiogram (ECG) or decreased pulmonary function during screening procedures.

**TABLE 1:** Characteristics of study participants

Characteristic	Value
Age (years)	38.3 ± 12.4
Body weight (kg)	73.1 ± 12.9
BMI (kg/m^2^)	27.5 ± 4.6
Hct (%)	70.6 ± 5.3
[Hb] (g/dl)	23.6 ± 1.8
CMS score	8.8 ± 3.9
$S_{pO_{2}}$ (%)	82.6 ± 3.5
HR (bpm)	81.9 ± 11.3
SBP (mmHg)	111.9 ± 8.9
DBP (mmHg)	77.1 ± 7.0

All values are presented as means ± SD. Abbreviations: BMI, body mass index; DBP, diastolic blood pressure; [Hb], haemoglobin concentration; Hct, haematocrit; HR, heart rate; $S_{pO_{2}}$, peripheral oxygen saturation; SBP, systolic blood pressure.

### 2.3 Preliminary screening, haematocrit and Qinghai CMS score

Clinical examination was performed during a preliminary screening session and general health information was collected. During this session, an ECG (Quark C12x, Cosmed, Albano Laziale, Italy) and spirometry (Pony FX, Cosmed, Albano Laziale, Italy) were performed, and pulse O_2_ saturation ($S_{pO_{2}}$) and heart rate (HR) were measured using a Nellcor N‐560 oximeter (Nellcor Puritan Bennet Inc., Pleasanton, CA, USA), and systolic and diastolic blood pressure (SBP and DBP, respectively) using a validated oscilometric device (UA‐767Plus, A&D, Tokyo, Japan; Verdecchia et al., 2004).

For screening purposes, haematocrit was determined from duplicate micro‐centrifuged blood samples obtained from a fingertip capillary blood draw. Participants with haematocrit ≥63% (equivalent to [Hb] ≥ 21 g/dl) were classified as individuals with EE according to the international consensus for high‐altitude chronic diseases and Qinghai score for CMS questionnaires that were used (Leon‐Velarde et al., 2005). CMS score determines the absence or presence and severity of the syndrome, and is based on the occurrence of EE and the presence and severity of the following signs and symptoms: headache, shortness of breath or palpitations, sleep disturbances, paresthesia, cyanosis, dilated veins, and tinnitus. CMS was diagnosed if the score was ≥6 (Leon‐Velarde et al., 2005).

### 2.4 Blood samples

Two 9‐ml blood samples were taken from the antecubital vein of all participants under fasting conditions on the morning of the preliminary cardiopulmonary exercise test (CPET) at baseline and after 4 and 8 weeks of ET. Blood samples were collected in clot‐activator and EDTA‐coated tubes. A micro‐capillary sample was obtained from the latter to determine venous haematocrit by centrifugation at 16,000 g for 10 min. Samples for blood analyses were centrifuged at 2750 g for 20 min to obtain serum or plasma, which were immediately stored at −20°C, and then at −80°C for storage and later analysis. Glucose, insulin, lipid profile, erythropoietin (EPO), and iron profile were measured in serum samples obtained after 10–12 h of fasting (MedLab Clinical Laboratory, Lima, Peru) at baseline and after 8 weeks of ET. The concentration of free plasma haptoglobin as a proxy for haemolysis was measured by enzyme‐linked immunosorbent assay according to the manufacturer's recommendations (Human Haptoglobin ELISA Kit ab108856, Abcam, Cambridge, UK) at baseline and after 4 and 8 weeks of ET. The homeostatic model assessment for insulin resistance (HOMA‐IR) index was calculated using fasting glucose and insulin values (HOMA2 Calculator v2.2.3, University of Oxford).

### 2.5 Ambulatory blood pressure monitoring

Ambulatory blood pressure monitoring (ABPM) is currently recognised by international guidelines as a key instrument for out‐of‐office blood pressure (BP) measurement and in the diagnosis and management of hypertension (Whelton et al., 2018; Williams et al., 2018). Available evidence indicates that ABPM may be superior to conventional office BP measurements allowing a more accurate assessment of the actual daily life BP (Bilo et al., 2020; Parati et al., 2008).

Twenty‐four‐hour ABPM was performed using a validated oscillometric device (TM‐2430; A & D; Palatini et al., 1998) applied on the non‐dominant arm at baseline and after 8 weeks of ET. Measurements took place every 15 min during daytime (06.00–23.00 h) and every 20 min during night‐time (23.00–06.00 h). Participants were asked to stay still during the recordings and keep a standardised activity journal. Valid ABPM recordings were those with at least 70% of expected readings available and which did not contain two or more consecutive hours without valid readings. Variables obtained from the recordings were systolic, diastolic and mean daytime (awake), night‐time (sleep) and 24‐h blood pressure. ABPM thresholds for hypertension were 135/85 mmHg for daytime, 120/70 mmHg for night‐time and 130/80 mmHg for 24 h (Whelton et al., 2018; Williams et al., 2018).

### 2.6 Cardiopulmonary exercise test

Participants performed a maximal cardiopulmonary exercise test (CPET) in Cerro de Pasco, Peru (4340 m) according to the guidelines of the American Thoracic Society/American College of Chest Physicians (2003). Earlobe and finger oximeter probes (Nellcor N‐560 oximeter, Nellcor Puritan Bennet Inc.) were used to obtain $S_{pO_{2}}$ measurements throughout the test. A tight‐fitting silicone oro‐nasal facemask (V2 series 7450 TM, Hans Rudolph, Shawnee, KS, USA) was secured and connected to an ergoespirometer‐metabolic system (Quark CPET, Cosmed, Albano Laziale, Italy). CPET was performed on a cycle ergometer (Ergomedic 828E, Monark Exercise AB, Vansbro, Sweden) with continuous breath‐by‐breath measurements of respiratory parameters to determine O_2_ consumption (${\dot{V}}_{O_{2}}$), CO_2_ production (${\dot{V}}_{CO_{2}}$), minute ventilation (${\dot{V}}_{E}$), and end‐tidal $P_{O_{2}}$ and $P_{CO_{2}}$ ($P_{\mathrm{ET}O_{2}}$ and $P_{\mathrm{ETC}O_{2}}$, respectively). Twelve‐lead ECG and HR were recorded continuously (Quark C12x, Cosmed, Albano Laziale, Italy). SBP and DBP during exercise was measured non‐invasively using sphyngomanometry at rest and then during the final minute of each workload increment. Participants performed a maximal preliminary CPET with a step‐incremental protocol at the beginning of the study to determine their aerobic capacity or peak ${\dot{V}}_{O_{2}}$ (${\dot{V}}_{O_{2}\mathrm{peak}}$). In a later session, a baseline five‐step CPET at 25%, 50%, 75% and 100% of each participant's pre‐determined ${\dot{V}}_{O_{2}\mathrm{peak}}$ was performed. All measurements were repeated after 4 and 8 weeks of ET.

### 2.7 Sub‐maximal aerobic exercise training

The training scheme consisted of sessions every other day with 2 days in a row weekly (4 days/week) to complete 32 sessions in 8 weeks. The protocol allowed one session/week as the maximum number of absences. Sub‐maximal aerobic ET consisted of pedalling exercise in a cycle‐ergometer at 60% of ${\dot{V}}_{O_{2}\mathrm{peak}}$ for 1 h/day. After 4 weeks, a maximal CPET was performed to readjust 60% of ${\dot{V}}_{O_{2}\mathrm{peak}}$ for the following 4 weeks. Training sessions were supervised by research personnel to ensure a proper workload (load and cadence) and duration. Each participant had a resting period of ∼48 h before performing CPET, and the day of the test counted as a training session.

$S_{pO_{2}}$, HR and in‐office SBP and DBP were measured in duplicate after 5 min of rest once the participant was seated on the cycle ergometer before each session and immediately after. No modification of diet or additional exercise activity was advised.

### 2.8 Sample size and statistical analysis

Assuming comparable differences and corresponding large effect sizes previously observed in haematocrit (*η* ^2^ = 0.72) and CMS score (*η* ^2^ > 1) in studies of CMS management with acetazolamide (Richalet et al., 2008), our primary end‐outcome variables after 8 weeks of ET in the present study required a sample size of eight participants in order to achieve a power of 0.80 at *P* < 0.05.

After testing for normality of data, repeated‐measures one‐way ANOVA or Friedman's test followed by Tukey's or Dunn's multiple comparisons, respectively, were performed. In the case of ABPM and blood markers, comparisons (baseline vs. 8 weeks) were made using Student's paired *t*‐test or Wilcoxon matched‐pairs signed rank test. All statistical tests were performed using GraphPad Prism version 9.1.2 for Windows (GraphPad Software, San Diego, CA, USA). Values are presented as means ± SD throughout.

## 3 RESULTS

### 3.1 General clinical screening

Table 1 shows the general characteristics of study participants. All showed typical CMS haematocrit and Qinghai score values (Leon‐Velarde et al., 2005), as well as marked hypoxaemia.

### 3.2 Sub‐maximal aerobic training sessions

Compliance to the training schedule, defined as the frequency of attendance to supervised ET sessions, had a rate of 96.8% (3.8 ± 0.27 sessions/week or 30.3 ± 0.97 out of 32 sessions/8 weeks). Before and after each ET session, $S_{pO_{2}}$ and HR at rest remained unchanged throughout the full 8‐week ET period compared to baseline measurements (*P* = 0.94). After 4 weeks, training workload was adjusted from its baseline value of 100 ± 15 to 118 ± 15 W to maintain 60% of maximal effort. There was no significant change in body weight or body mass index.

### 3.3 Haematocrit and Qinghai CMS score

**FIGURE 1:**
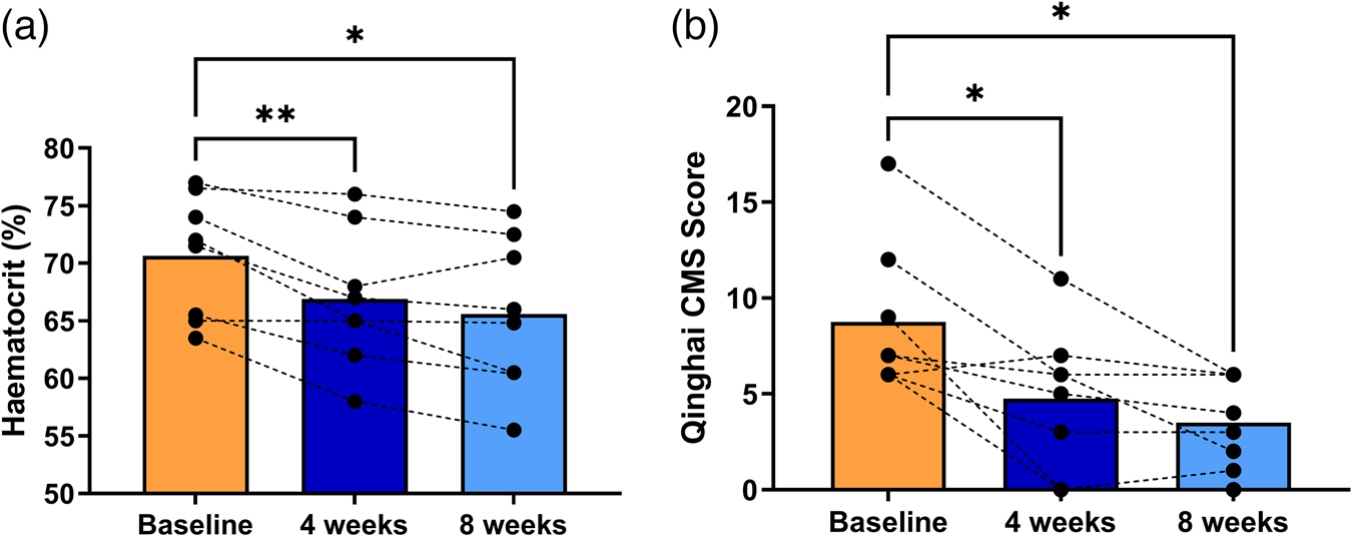
Effect of aerobic exercise training on haematocrit and CMS score. (a) Haematocrit at baseline and after 4 and 8 weeks of ET in highlanders with CMS. (b) The same comparison for the Qinghai CMS score. Bars represent means, and repeated measures of each participant are connected by dashed lines. Changes in haematocrit and CMS score were assessed employing repeated measures one‐way ANOVA and Tukey's multiple comparison test. *P < 0.05, **P < 0.01 vs. baseline

Baseline venous haematocrit (70.6 ± 5.3%) decreased significantly by 5% and 7% (*P *< 0.05) on week 4 (to 66.8 ± 5.9%) and 8 (to 65.6 ± 6.6%), respectively, while Qinghai CMS score also showed a significant reduction to 4.8 ± 1.3 and 3.5 ± 0.8, respectively (*P *< 0.05) (Figure 1).

### 3.4 Cardiopulmonary exercise test

Ambient conditions during CPET sessions were stable with barometric pressure of 456 ± 0.3 mmHg, mean room temperature of 20 ± 0.3°C and relative humidity of 61 ± 0.7%.

Table 2 shows CPET measurements together with $S_{pO_{2}}$ and BP values at rest before the incremental exercise protocol and during peak exercise at baseline and after 4 and 8 weeks.

**TABLE 2:** Cardiopulmonary exercise test

Parameter	Baseline	4 weeks	8 weeks
Rest			
${\dot{V}}_{O_{2}}$ (ml/kg/min)	4.55 ± 0.6	5.05 ± 0.9	4.69 ± 1.0
${\dot{V}}_{CO_{2}}$ (ml/kg/min)	4.62 ± 0.7	5.21 ± 1.1	4.58 ± 1.2
${\dot{V}}_{E}$ (l/kg/min)	0.17 ± 0.03	0.19 ± 0.1	0.17 ± 0.03
$P_{\mathrm{ETC}O_{2}}$ (mmHg)	31.2 ± 2.3	31.5 ± 1.8	29.5 ± 2.2
$P_{\mathrm{ET}O_{2}}$ (mmHg)	53.6 ± 3.3	53.2 ± 2.3	54.2 ± 1.1
*R*_f_ (bpm)	17.6 ± 2.1	17.5 ± 2.4	17.1 ± 2.7
HR (bpm)	81.9 ± 11.3	77.7 ± 8.5	72.1 ± 2.8
$S_{pO_{2}}$ (%)	84.3 ± 2.8	85.2 ± 3.3	85.4 ± 2.1
SBP (mmHg)	115.8 ± 10.8	110.1 ± 11.1	109.8 ± 14.6
DBP (mmHg)	70.6 ± 8.1	67.5 ± 7.1	66.1 ± 9.6
Peak exercise			
${\dot{V}}_{O_{2}}$ (ml/kg/min)	33.8 ± 6.7	36.4 ± 5.4	37.2 ± 5.6*
${\dot{V}}_{CO_{2}}$ (ml/kg/min)	40.7 ± 7.4	42.2 ± 5.7	42.6 ± 5.8
${\dot{V}}_{E}$ (l/kg/min)	1.8 ± 0.5	1.9 ± 0.5	2.0 ± 0.4
$P_{\mathrm{ETC}O_{2}}$ (mmHg)	24.8 ± 4.1	24.4 ± 2.8	23.6 ± 2.5
$P_{\mathrm{ET}O_{2}}$ (mmHg)	63.3 ± 3.0	63.0 ± 2.0	63.1 ± 1.5
*R*_f_ (bpm)	52.3 ± 8.8	57.4 ± 9.6	56.6 ± 6.8
HR (bpm)	153.4 ± 22.4	164.9 ±19.1	169.5 ± 16.5*
$S_{pO_{2}}$ (%)	80.1 ± 3.5	81.8 ± 3.9	79.6 ± 2.4
SBP (mmHg)	156.3 ± 17.5	140.0 ± 10.4**	148.3 ± 15.8
DBP (mmHg)	89.3 ± 2.9	81.4 ± 5.9*	86.0 ± 4.8
Workload (W)	172.5 ± 26.6	198.8 ± 27.5***	210.0 ± 27.8**

All values are presented as means ± SD. Differences were assessed using repeated measures one‐way ANOVA or Friedman tests followed by Tukey's or Dunn's multiple comparisons, respectively.

^*^ *P *< 0.05, ^**^ *P *< 0.01 vs. baseline. Abbreviations : ${\dot{V}}_{CO_{2}}$, CO_2_ production; ${\dot{V}}_{O_{2}}$, O_2_ consumption; ${\dot{V}}_{E}$, pulmonary ventilation; $P_{\mathrm{ETC}O_{2}}$, end‐tidal CO_2_ partial pressure; $P_{\mathrm{ET}O_{2}}$, end‐tidal O_2_ partial pressure; *R*_f_, respiratory rate; HR, heart rate; $S_{pO_{2}}$, peripheral oxygen saturation; SBP, systolic blood pressure; DBP, diastolic blood pressure.

**FIGURE 2:**
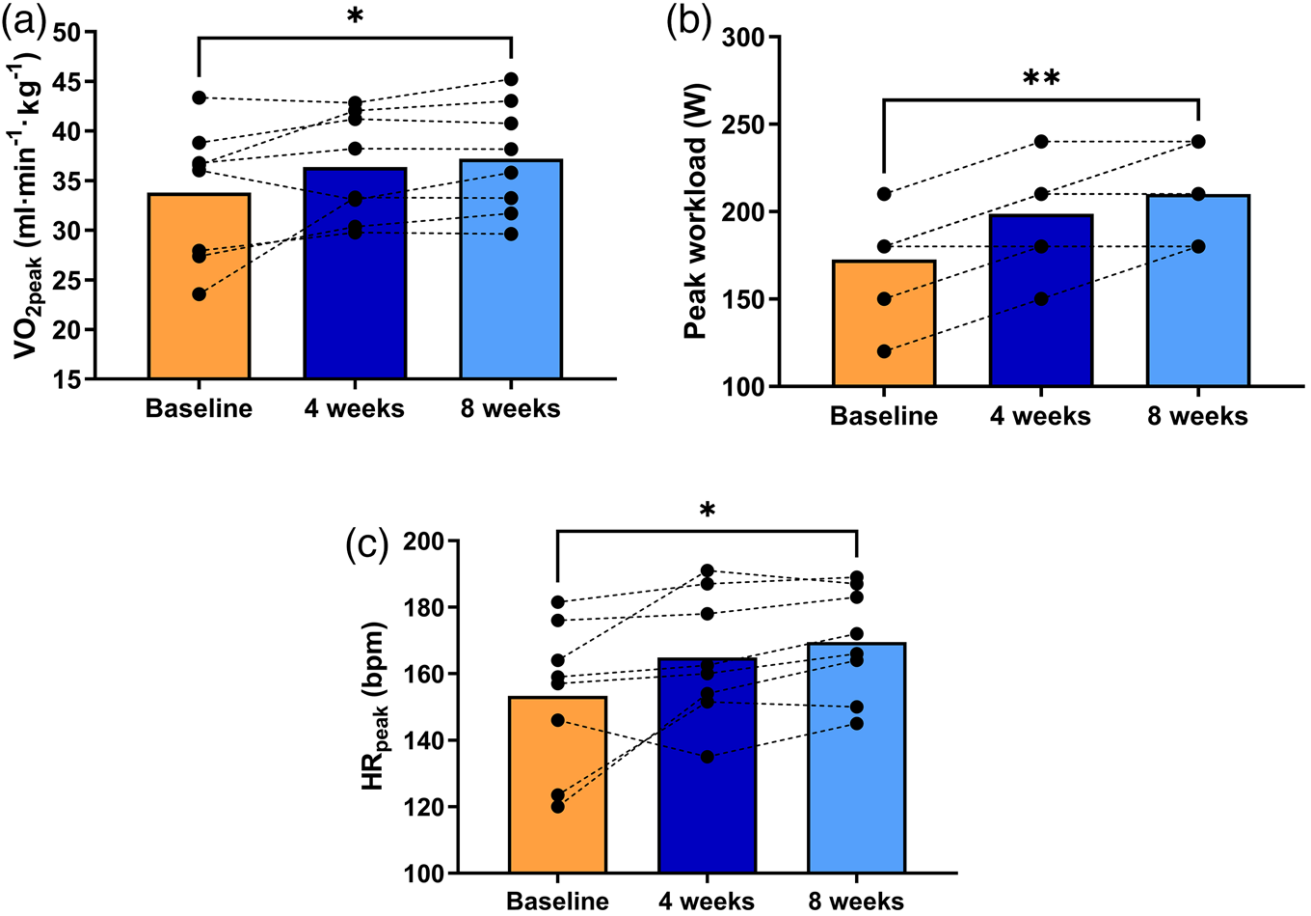
Effect of exercise training on aerobic capacity, peak workload and peak heart rate. (a) Aerobic capacity (${\dot{V}}_{O_{2}\mathrm{peak}}$) at baseline and after 4 and 8 weeks of ET in highlanders with CMS. (b) The corresponding measurements of baseline peak workload (W_peak_) and its change after 4 and 8 weeks of ET. Individual values overlap due to similar workload of participants at each time point. (c) The same comparison of baseline, 4 and 8 weeks for peak HR (HR_peak_). Bars represent means, and repeated measures of each participant are connected by dashed lines. Changes in ${\dot{V}}_{O_{2}}$, workload and HR at peak exercise were assessed employing repeated measures one‐way ANOVA and Tukey's multiple comparison test. *P < 0.05, **P < 0.01 vs. baseline

After 4 weeks, a maximal exercise test was repeated to readjust 60% of ${\dot{V}}_{O_{2}\mathrm{peak}}$ for the following 4 weeks. ${\dot{V}}_{O_{2}\mathrm{peak}}$ increased after 8 weeks (33.8 ± 2.4 vs. 37.2 ± 2.0 ml/min/kg, *P *< 0.05; Figure 2a), and maximum baseline workload (172.5 ± 9.4 W) also increased significantly at week 4 and 8 (198.9 ± 9.7 and 210.0 ± 27.8 W, *P *< 0.01, respectively; Figure 2b). Peak HR (HR_peak_) showed an increase at week 8 compared to baseline values (Table 2 and Figure 2c). While at rest SBP and DBP showed no change over 8 weeks of ET, both showed a significant fall at peak exercise at week 4 (*P *< 0.05), but no statistically significant reduction after 8 weeks.

### 3.5 Ambulatory blood pressure monitoring

ABPM results are shown in Table 3. ABPM parameters showed no differences after 8 weeks of aerobic ET compared to baseline measurements.

**TABLE 3:** Ambulatory blood pressure monitoring

Parameter	Baseline	8 weeks
24 h		
SBP (mmHg)	115.3 ± 6.6	120.1 ± 9.2
DBP (mmHg)	71.7 ± 3.7	74.4 ± 3.5
MAP (mmHg)	85.9 ± 4.3	89.1 ± 5.3
HR (bpm)	76.7 ± 5.5	76.1 ± 5.5
Daytime		
SBP (mmHg)	117.0 ± 5.8	123.0 ± 10.8
DBP (mmHg)	73.4 ± 4.0	76.4 ± 5.1
MAP (mmHg)	87.7 ± 3.9	91.9 ± 6.5
HR (bpm)	76.4 ± 8.5	75.9 ± 7.8
Sleep		
SBP (mmHg)	105.4 ± 11.8	104.4 ± 13.7
DBP (mmHg)	63.4 ± 7.4	65.1 ± 6.0
MAP (mmHg)	77.0 ± 8.5	77.9 ± 8.4
HR (bmp)	72.9 ± 9.2	71.6 ± 10.0

All values are presented as means ± SD. Comparisons were made using paired *t*‐test or Wilcoxon matched‐pairs signed rank test. Abbreviations: DBP, diastolic blood pressure; HR, heart rate; MAP, mean arterial blood pressure; SBP, systolic blood pressure.

### 3.6 Cardiometabolic risk, erythropoietic and haemolysis blood markers

Average glycaemia, insulinaemia or HOMA‐IR index values, as well as EPO and iron profile markers, showed no differences after 8 weeks of ET. Lipid profile analyses showed no differences except for an increase in high‐density lipoprotein cholesterol (HDL‐C) at the end of the study (Table 4).

**TABLE 4:** Cardiometabolic risk and erythropoietic blood markers

Cardiometabolic risk blood markers	Baseline	8 weeks
Glucose (mg/dl)	101.8 ± 28.5	96.4 ± 18.4
Insulin (μIU/ml)	10.3 ± 6.7	10.4 ± 9.5
HOMA‐IR (%)	1.5 ± 0.8	1.4 ± 1.1
Total cholesterol (mg/dl)	166.1 ± 45.9	161.4 ± 49.2
HDL‐C (mg/dl)	39.0 ± 9.3	43.8 ± 9.9*
LDL‐C (mg/dl)	82.6 ± 49.9	89.2 ± 45.6
Triglycerides (mg/dl)	222.8 ± 160.3	142.4 ± 62

Erythropoietic blood markers		
EPO (mIU/ml)	14.31 ± 8.0	20.43 ± 14.6
Iron (mg/dl)	108.4 ± 29.6	120.7 ± 38.1
Ferritin (mg/dl)	89.3 ± 64.9	112.5 ± 90.1
Transferrin (mg/dl)	305.1 ± 30.0	302.9 ± 25.8

All values are presented as means ± SD. Comparisons were made using paired *t*‐test or Wilcoxon matched‐pairs signed rank test.

* *P *< 0.05 vs. baseline. Abbreviations: EPO, erythropoietin; HDL‐C, high‐density lipoprotein cholesterol; HOMA‐IR, homeostatic model assessment for insulin resistance; LDL‐C, low‐density lipoprotein cholesterol.

**FIGURE 3:**
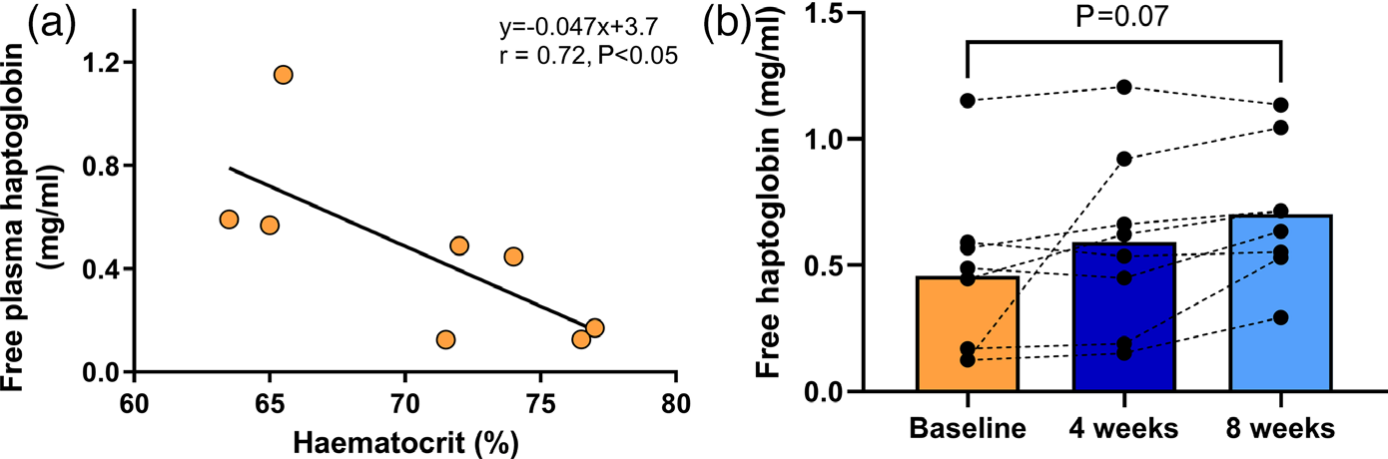
Effect of exercise training on free plasma haptoglobin. (a) The significant inverse correlation between free plasma haptoglobin and haematocrit in highlanders with CMS at baseline. (b) Free plasma haptoglobin at baseline and after 4 and 8 weeks of ET with a tendency to increase in the latter (*P* = 0.07). Bars represent means, and repeated measures of each participant are connected by dashed lines. Levels of free plasma haptoglobin were assessed employing repeated measures one‐way ANOVA and Tukey's multiple comparison test

At baseline, free haptoglobin showed a negative correlation with haematocrit (*r *= 0.72, *P *< 0.05, Figure 3a) which lost statistical significance after 4 (*r *= −0.65, *P* = 0.08) and 8 (*r *= −0.51, *P* = 0.20) weeks of ET once haematocrit decreased. Free plasma haptoglobin concentration showed a non‐significant trend to increase from baseline to week 8 (*P* = 0.07, Figure 3b).

## 4 DISCUSSION

We showed that sub‐maximal aerobic ET for 8 weeks reduces haematocrit and ameliorates CMS signs and symptoms. Despite some evidence regarding diminished exercise capacity (Winslow & Monge‐C, 1987; Winslow et al., 1985), or exercise being counterproductive for highlanders with CMS (Pratali et al., 2012; Soria et al., 2019; Stuber et al., 2010), we show that an exercise programme at 60% of maximal effort for 4 days a week during 8 weeks is a potential management approach for CMS.

Bloodletting and haemodilution have been the traditional management strategies for CMS at high altitude (Klein, 1983; Sedano et al., 1988; Winslow & Monge‐C, 1987; Winslow et al., 1985). Interestingly, signs and symptoms resume within hours when haematocrit is reduced by these methods despite environmental hypoxia, showing that CMS symptomatology is secondary to EE. Pharmacological treatment strategies have been used to reduce haematocrit either by reducing the erythropoietic stimulus or erythropoiesis itself. These interventions included angiotensin converting enzyme inhibitors (Plata et al., 2002; Vargas et al., 1996), dopaminergic antagonists (Leon‐Velarde et al., 2003) and ventilatory stimulants such as medroxyprogesterone (Kryger et al., 1978) and almitrine (Villena et al., 1985). However, only a few studies have shown evidence for safety and efficacy in the treatment of CMS. The most recent and longer‐term randomised controlled trials with clinical significance used acetazolamide, a systemic carbonic anhydrase inhibitor, as a potential treatment of the syndrome. Two randomised, double‐blind, placebo‐controlled studies assessed the safety and efficacy of acetazolamide treatment for up to 6 months in CMS patients in Cerro de Pasco, Peru (Richalet et al., 2008; Richalet et al., 2005). Results showed that acetazolamide increased $P_{aO_{2}}$, decreased serum EPO and decreased haematocrit by 5%. Treatment also decreased pulmonary vascular resistance, increased nocturnal $S_{pO_{2}}$ and reduced sleep‐disordered breathing episodes. Overall, acetazolamide reduced hypoventilation, blunted erythropoiesis and improved pulmonary circulation without adverse effects throughout the duration of the trials. Although its implementation as a treatment appears efficient and safe, longer trials would be required to assess any development of tolerance or potential long‐term consequences of the chronic inhibition of carbonic anhydrase in the different organs and systems. For this reason, non‐pharmacological approaches are an interesting avenue to explore, especially in resource‐constrained populations.

Native highlanders who exercise regularly, or highly trained highlander athletes, have significantly lower haematocrit values compared to the sedentary population at the same altitude. We showed that 8‐week submaximal aerobic ET reduced haematocrit by 7% and ameliorated CMS signs and symptoms significantly, reducing CMS score down to values usually observed in the healthy highlander population. Our results paralleled those obtained with acetazolamide treatment, but without the improvement on $S_{pO_{2}}$. In addition, we did not detect any significant change in $P_{\mathrm{ET}O_{2}}$ or $P_{\mathrm{ETC}O_{2}}$. The reduction of haematocrit might be explained by the expansion of plasma volume (PV) and the consequent haemodilution, as this has been well documented in both cross‐sectional and longitudinal endurance ET studies (Convertino, 2007; Schmidt & Prommer, 2008). PV expansion can account for nearly all of the ET‐induced hypervolaemia up to 2–4 weeks; after this time expansion may be distributed equally between plasma and red cell volumes (Convertino, 2007). Hypervolaemia may provide larger vascular volume and filling pressure for greater cardiac stroke volume, and thus cardiac output ($\dot{Q}$) during exercise. This, together with greater ET‐induced O_2_ extraction and increased muscle mitochondrial oxidative capacity (Skattebo et al., 2020) might also contribute to the increased aerobic capacity after 8 weeks of ET despite decreased haematocrit and unchanged $S_{pO_{2}}$. In addition, we have recently shown that aerobic capacity is supported by adrenergic and non‐adrenergic vasoconstriction of non‐active skeletal muscle in individuals with CMS, which likely aids in central redistribution of blood volume during exercise (Hansen et al., 2021). Also, heightened α‐adrenergic signalling restrains vasodilatation within active skeletal muscle to better match O_2_ delivery (Hansen et al., 2021).

Interestingly, lower haematocrit (lower Hb concentration) might also contribute to increased O_2_ diffusion and O_2_ extraction between muscle microcirculatory vessels and mitochondria (Wagner, 1996). When Hb concentration is reduced, time to diffusive equilibration in these vascular beds is shortened. Piiper & Scheid (1981) showed that the compound constant *D*/(β$\dot{Q}$) determined the degree of diffusion equilibration to be expected in muscle tissue, where *D* is muscle diffusion coefficient and β is the (average) slope of the O_2_–Hb dissociation curve. As β must decrease when total Hb concentration decreases, *D*/(β$\dot{Q}$) must rise, even taking into account a modest increase in $\dot{Q}$, diffusion equilibration would take less time. As a result muscle O_2_ extraction increases when Hb concentration is lower, and overall O_2_ transport is improved, counterbalancing the effect of decreased arterial O_2_ content.

ET‐induced PV and blood volume (BV) expansion together with the reduction of haematocrit are also associated with increased serum EPO levels. Montero et al. (2017) studied the changes in PV, BV and overnight fasting haematological markers before and at 2, 4 and 8 weeks of supervised ET consisting of three to four 60‐min (50–60% of maximal power output) cycloergometry sessions per week for 8 weeks. Increases in PV and BV were observed in week 2 (+16%) and remained stable through week 8 (+14%). Total red blood cell volume (RBCV) increased by 6% in week 4 and 12% by week 8, while overall BV increased by 13% from week 0 to 8. Hematocrit decreased 3% (−7%) in week 2 and remained somewhat reduced at week 8 (−3%). This reduction is slightly less to what we found in our study after 4 (−5%) and 8 weeks (−7%) of ET possibly because of the much higher haematocrit of CMS highlanders which might favour haemolysis due to mechanical stress. Montero and colleagues also showed that overnight‐fasting EPO concentration increased by 25% in week 2, and was no longer different from baseline (week 0) at week 4 and 8. Our results shows that serum EPO exhibited no significant differences at week 4 and 8 compared to baseline, suggesting that we might have lost the earlier window (i.e., 2 weeks) where ET‐induced plasma expansion and serum EPO are maximal. Nevertheless, the fact that EPO remains unaltered during 8 weeks of training suggest that the erythropoietic stimulus is minor. Overall, despite ET‐induced increased RBCV, plasma expansion overrides any increase in red blood cell mass and results in reduced haematocrit. In the case of CMS highlanders this reduction can be magnified by ET‐induced mechanical haemolysis due to excessive haematocrit levels (Macarlupu et al., 2021; Telford et al., 2003). In fact, we have recently shown that haemolysis might be an additional mechanism for ET‐induced haematocrit reduction in a rat model of high‐altitude erythrocytosis (Macarlupu et al., 2021). Haemolysis due to mechanical stress might lead to a reduction in haematocrit without significant modification of PV or total blood volume (Schobersberger et al., 1990; Selby & Eichner, 1986; Telford et al., 2003). This mechanism is particularly expected to be relevant in impact sports such as running. However, there is also evidence of haemolysis induced by exercise in disciplines where impact is reduced or absent (Lippi & Sanchis‐Gomar, 2019; Schobersberger et al., 1990; Selby & Eichner, 1986). Haemolysis would occur to a greater degree when blood shows a higher viscosity, and therefore it would be favoured when haematocrit is excessive. As haptoglobin binds and combines with free plasma Hb, our finding of an inverse relationship of free haptoglobin and haematocrit at baseline suggests a steady‐state background haemolysis due to EE. However, this relationship is lost after 4 and 8 weeks of ET once haematocrit decreased, and total plasma free haptoglobin showed a trend to rise suggesting a marginal increased haemolysis consequent to ET.

One other important effect of ET is the reduction of BP. Studies at sea‐level have shown that aerobic ET for 8 weeks (30‐min session; 3 times/week at 80–90% of ventilatory threshold) decreases 24‐h SBP and DBP, with the largest reduction mainly at night‐time (Carvalho et al., 2009). However, our results show no reduction in office‐based BP or ABPM at rest after 8 weeks of aerobic ET. This lack of change might be explained by a possible concurrent effect of the autonomic characteristics of CMS highlanders, the ET‐induced expansion of PV and the ET‐induced drop in peripheral vascular resistance (Fagard, 2006). We have recently shown that in comparison with healthy Andean highlanders, CMS individuals show lower basal sympathetic vasoconstrictor drive with enhanced cardiovagal baroreflex gain (Moore et al., 2006; Simpson et al., 2021), which might compensate for the haemodynamic consequences of EE and a possible ET‐induced expanded blood volume. Such changes appear to be adaptive physiological responses to the elevated haematocrit, which allow BP homeostasis to be maintained. We have also shown that Andeans with mild‐to‐moderate erythrocytosis have a substantial reduction in the peripheral vascular resistance given the prevailing sympathetic activity, which is likely related to their lower basal vascular tone. Ultimately, vascular phenotypic adaptations in CMS highlanders, in both conduit artery and downstream arterioles, allow the maintenance of local and total vascular resistances, resting BP and vascular shear patterns (Hansen et al., 2021). This effect is probably augmented by ET as we observed a significant drop in SBP and DBP at peak exercise after 4 weeks. Nevertheless, the expression of this phenotype is possibly modest and might be lost over time (Bilo et al., 2020; Corante et al., 2018).

A compensatory or offsetting effect on HR might be also taking place during ET. Our results show that although not statistically significant, HR at rest shows a trend of continuous reduction after 8 weeks of ET compared to baseline values. Normally, ET induces a reduction in HR and the effect occurs after only a few months, with about three training sessions per week (Genovesi et al., 2007; Whyte et al., 2008; Zavorsky, 2000). Meta‐analyses on the effect of ET over HR indicate that endurance training decreases the resting HR between 4 and 6 bmp (Huang et al., 2005; Reimers et al., 2018). Similarly, HR_peak_ is reduced following regular aerobic exercise in sedentary adults and endurance athletes. The overall effect of aerobic training on HR_peak_ is moderate as it can be altered by 3–7% (Zavorsky, 2000). It is possible that severe hypoxaemia and basal enhanced sympathetic activity in CMS highlanders prevent the full reduction of resting HR in CMS and may lead to augment HR at peak exercise.

Lastly, aerobic ET is also associated to improved cardiometabolic risk profile (Lin et al., 2015). In previous studies we have identified independent associations between EE and 24 h ambulatory hypertension including systolic–diastolic and isolated diastolic hypertension (Bilo et al., 2020; Corante et al., 2018), and a significant proportion of masked hypertension, the latter linked to increased cardiovascular morbidity and mortality in lowlanders (Pierdomenico & Cuccurullo, 2011). We have also identified independent associations between EE, insulin resistance, hyperglycaemia, and dyslipidaemia. These findings agree with other studies at high altitude in the Peruvian Andes that have identified independent relationships between EE and hypertension, hypertriglyceridaemia (Gonzales & Tapia, 2013; Jefferson et al., 2002; Leon‐Velarde & Arregui, 1994) and metabolic syndrome (De Ferrari et al., 2014). Several RCTs have shown reductions in blood triglycerides, glucose and insulin, and increases in HDL‐C as the most common biomarker changes following ET programmes (Lin et al., 2015). Circulating HDL‐C levels are usually significantly increased with ET in a manner that is dependent on the duration and intensity of the training plan (Durstine et al., 2001; Kodama et al., 2007). Our results showed a significant increase in HDL‐C, a trend to triglyceride reduction and no changes in glycaemic control markers. Studies with training plans from 4 to 104 weeks with mild or moderate intensity reported changes in some cardiometabolic marker levels (Lin et al., 2015), such as an increase in HDL‐C even as early as 4 weeks of ET (Banz et al., 2003; Cox et al., 1993; Haskell, 1984; Kraus et al., 2002; LeMura et al., 2000). On the other hand, other blood lipid, as well as glycaemic control markers, frequently tend to improve over a period ≥12 weeks of ET (Cho et al., 2011; Libardi et al., 2012; Watkins et al., 2003) suggesting that possibly the length of our study was not enough to observe such changes.

### 4.1 Limitations

One main limitation of our study is that although it is difficult to maintain a group of participants training for 8 weeks, the protocol might have been controlled with the inclusion of CMS highlanders without ET and/or a group of non‐CMS highlanders subjected to a similar ET programme. Either of the two comparisons would have been interesting and informative. However, we believe that this does not preclude the conclusions of our study as each participant was their own baseline control and the main aim of the study was to investigate whether ET can reduce haematocrit and alleviate symptoms in CMS highlanders.

The inclusion of male highlanders only is an additional limitation of our study. CMS has a very low prevalence in pre‐menopausal women (Azad et al., 2021; Leon‐Velarde et al., 1997; Leon‐Velarde et al., 2001), and therefore forming comparable groups covering the same age range to avoid any confounding effects of age, and of menopause itself, would have been difficult. For these and cultural reasons, women were not included in the study and therefore our findings cannot be extrapolated to female CMS highlanders.

### 4.2 Conclusions

In conclusion, we show that sub‐maximal aerobic ET can effectively reduce haematocrit and ameliorate symptoms in CMS patients. In addition, despite the common belief of reduced tolerance to exercise in CMS highlanders, we show that ET can successfully improve aerobic capacity and exercise workload in these patients maintaining BP homeostasis and improving cardiovascular disease risk markers such as HDL‐C. Overall, our results suggest that a regular aerobic ET might be used as a low‐cost non‐invasive and non‐pharmacological practical management strategy for CMS.

## COMPETING INTERESTS

The authors declare that the research was conducted in the absence of any commercial or financial relationships that could be construed as a potential conflict of interest.

## AUTHOR CONTRIBUTIONS

J.L.M. and F.C.V. conceived and designed the research; R.F.‐M., G.V.‐G., J.L.M. and F.C.V. performed the experiments and analysed the data; J.L.M. and F.C.V. interpreted results of the experiments, prepared the figures and drafted the manuscript; J.L.M., R.F.‐M., G.V.‐G., J.‐P.R., N.V. and F.C.V. edited and revised the manuscript. All authors have read and approved the final version of this manuscript and agree to be accountable for all aspects of the work in ensuring that questions related to the accuracy or integrity of any part of the work are appropriately investigated and resolved. All persons designated as authors qualify for authorship, and all those who qualify for authorship are listed.

## Supporting information

Statistical Summary Document

## Data Availability

Datasets used and/or analysed during the current study are available from the corresponding author on reasonable request.
